# Rehabilitation Response in Tremor‐ and Non‐Tremor‐Dominant Parkinson Disease: A Task‐fMRI Study

**DOI:** 10.1002/brb3.70102

**Published:** 2024-10-17

**Authors:** Keke Chen, Songjian Wang, Qiping Wen, Zhaohui Jin, Yixuan Wang, Detao Meng, Xin Yu, Mengyue Wang, Meng Lin, Youwei Li, Chunlin Li, Boyan Fang

**Affiliations:** ^1^ Parkinson Medical Center, Beijing Rehabilitation Hospital Capital Medical University Beijing China; ^2^ Beijing Institute of Otolaryngology, Otolaryngology—Head and Neck Surgery, Key Laboratory of Otolaryngology Head and Neck Surgery (Capital Medical University), Ministry of Education, Beijing Tongren Hospital Capital Medical University Beijing China; ^3^ School of Biomedical Engineering, Key Laboratory of Fundamental Research on Biomechanics in Clinical Application Capital Medical University Beijing China; ^4^ Radiology Department, Beijing Rehabilitation Hospital Capital Medical University Beijing China; ^5^ School of Beijing Rehabilitation Medicine Capital Medical University Beijing China

**Keywords:** brain activation, fMRI, functional connectivity, motor subtypes, Parkinson's disease, rehabilitation

## Abstract

**Background:**

Tremor‐dominant (TD) and nontremor‐dominant (NTD) Parkinson's disease (PD) showed different responses to rehabilitation. However, the neural mechanism behind this remains unclear.

**Methods:**

This cohort study explores changes in motor function, brain activation, and functional connectivity following 2 weeks of rehabilitation in TD‐PD and NTD‐PD patients, respectively. A total of 11 TD‐PD patients, 24 NTD‐PD patients, and 21 age‐matched healthy controls (HCs) were included. At baseline, all participants underwent functional magnetic resonance imaging (fMRI) while performing the foot tapping task. Motor symptoms, gait, balance, and task‐based fMRI were then evaluated in patients before and after rehabilitation.

**Results:**

Compared to HCs, TD‐PD patients showed increased activity in the left inferior frontal gyrus and the right insula, and NTD‐PD patients showed increased activations in the left postcentral gyrus and decreased within‐cerebellar connectivity at baseline. Rehabilitation improved motor function in PD patients regardless of motor subtype. TD‐PD patients showed increased recruitments of the sensorimotor cortex and the bilateral thalamus after rehabilitation, and NTD‐PD patients showed increased cerebellar activation and within‐cerebellar connectivity that was associated with better motor performance.

**Conclusions:**

This study demonstrated that rehabilitation‐induced brain functional reorganization varied by motor subtypes in PD, which may have important implications for making individualized rehabilitation programs.

**Trial Registration:**

ClinicalTrials.gov identifier: ChiCTR1900020771

## Graphical abstract

Changes of brain activation and functional connectivity were different in tremor‐dominant and nontremor‐dominant Parkinson's disease during foot tapping task after rehabilitation. TD‐PD patients showed increased recruitments of the sensorimotor cortex and the bilateral thalamus after rehabilitation, and NTD‐PD patients showed increased cerebellar activation and within‐cerebellar connectivity that was associated with better motor performance.

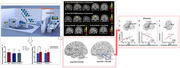

## 1 Introduction

The cardinal motor features of Parkinson's disease (PD) are resting tremor, bradykinesia, rigidity, and postural instability (Goetz 2011), and these symptoms can manifest as different clinical phenotypes. Two of the most common motor subtypes of PD are tremor dominant (TD) and non‐tremor dominant (NTD) (Jankovic et al. 1990). This motor subtyping may allow a greater understanding of the pathophysiology of PD and may lead to improvements in rehabilitation techniques as well. Tremor‐dominant PD (TD‐PD) is characterized by the presence of tremors and slow disease progression, but nontremor‐dominant PD (NTD‐PD) involves bradykinesia, rigidity, postural instability/gait difficulty (PIGD), rapid disease progression, and marked cognitive decline (Arie et al. 2017; Burn et al. 2006; Rajput et al. 2009). These two motor subtypes differ not only in clinical manifestation but also in neuroimaging results (Xiang et al. 2017). Studies suggest that NTD‐PD patients have deficits within the striato‐thalamo‐cortical circuit (Boonstra et al. 2021) and that resting tremor emerges from the cerebello‐thalamo‐cortical circuit when triggered by transient pathological signals from the basal ganglia motor loop (Helmich et al. 2012, 2011). In addition, TD and NTD subtypes respond differently to rehabilitation. Our previous study showed that NTD‐PD patients had greater improvement in motor symptoms than TD‐PD patients after short‐term multidisciplinary intensive rehabilitation (Chen et al. 2021). However, the neural mechanism behind this remains unclear.

Task‐based functional magnetic resonance imaging (fMRI), which collects data on the activation of task‐related brain regions, is a noninvasive and useful approach to evaluating brain activity changes for different conditions or after specific training (Filippi et al. 2018), and this method is helpful for understanding the effects of rehabilitation on neuroplasticity in patients with PD (PWPs) in particular. Using task‐based fMRI, studies have indicated that rehabilitation‐induced gains in PWPs are associated with increased recruitments of the brain's motor areas, attentional control areas, and the cerebellum (Agosta et al. 2017; Sarasso et al. 2021; Vieira‐Yano et al. 2021). Segura et al. suggested that the therapeutic effect of rehabilitation in PWPs may be driven by strengthening of thalamocortical connectivity (Segura et al. 2020). However, few studies have examined rehabilitation‐induced alterations of brain activation and functional connectivity (FC) in TD‐PD and NTD‐PD patients during a motor task. Considering that multidisciplinary intensive rehabilitation is useful in improving motor functions, especially gait and balance, in this study we chose the foot tapping task (Agosta et al. 2017; Ehgoetz Martens et al. 2018) to investigate rehabilitation‐induced changes in brain function of TD‐PD and NTD‐PD patients. Based on our previous research, this exploratory study aims to investigate alterations in motor function, brain activation, and FC following 2 weeks of rehabilitation in TD‐PD and NTD‐PD patients. We hypothesize that rehabilitation‐induced changes in brain activation and FC vary by motor subtypes.

## 2 Methods

### 2.1 Subjects and Study Design

For the present study, we conducted secondary analysis of the data from previous project no. ChiCTR1900020771 (Chen et al. 2021). Sixty‐nine idiopathic PWPs were recruited according to the Movement Disorder Society criteria (Postuma et al. 2015). The PWPs were subtyped into a TD group and an NTD group according to the method proposed by Stebbins et al. (2013). Twenty‐one age‐ and sex‐matched healthy controls (HCs) were also recruited and performed the same task‐based fMRI at baseline as the PWPs.

The inclusion consisted of the following: Hoehn–Yahr stage (Hoehn and Yahr 1967) < 4; dopaminergic medication without any changes within 3 months; and no cognitive impairment (division criteria of the Mini‐Mental State Examination [MMSE] scale) (Katzman et al. 1988). Participants were excluded if they had DBS or in vivo implantation treatment; osteoarthritis, lumbar disc herniation, or fractures that may affect walking ability; a history of any other severe brain disease, such as stroke or brain tumor; brain surgery; serious visual or hearing impairments; psychotic symptoms; or any contraindications for MRI.

### 2.2 Multidisciplinary Intensive Rehabilitation Treatment

All PWPs underwent the 2‐week rehabilitation program that we have reported previously (Chen et al. 2021). This program was carried out in a hospital setting, four sessions each day, 5 days per week. Briefly, the first session was 30‐min physical therapy; the second session was balance and gait training via the Balance Tutor (Meditouch, Netanya, Israel) and C‐MiLL (Motek, Amsterdam/Culemborg, Netherlands); the third session was aerobic training using a trainer (T5XR; Nustep, Ann Arbor, MI, USA); and the last session was speech therapy. Each session lasted 30–60 min.

### 2.3 Clinical Evaluations

The Hoehn–Yahr scale (Hoehn and Yahr 1967) and Movement Disorders Society Unified Parkinson's Disease Rating Scale Part III (MDS‐UPDRS III) (Goetz et al. 2008) were assessed by experienced neurologists. According to the cardinal symptoms, MDS‐UPDRS III were further subdivided into the following four subscales: tremor, bradykinesia, rigidity, and axial (Li et al. 2018). A MMSE was evaluated by a cognitive specialist, and the following motor functional evaluations were conducted by an experienced physiotherapist: the Berg Balance Scale (BBS) (Berg, Wood‐Dauphinee, and Williams 1995), the Timed Up and Go test (TUG) (Morris, Morris, and Iansek 2001), the 10‐Meter Walk Test (10MWT) (Lang et al. 2016), and the 6‐Minute Walk Test (6MWT) (ATS Committee on Proficiency Standards for Clinical Pulmonary Function Laboratories 2002). Levodopa equivalent doses (LEDs) (Tomlinson et al. 2010) were also recorded.

### 2.4 MRI

#### 2.4.1 Foot Tapping Task

**FIGURE 1:**
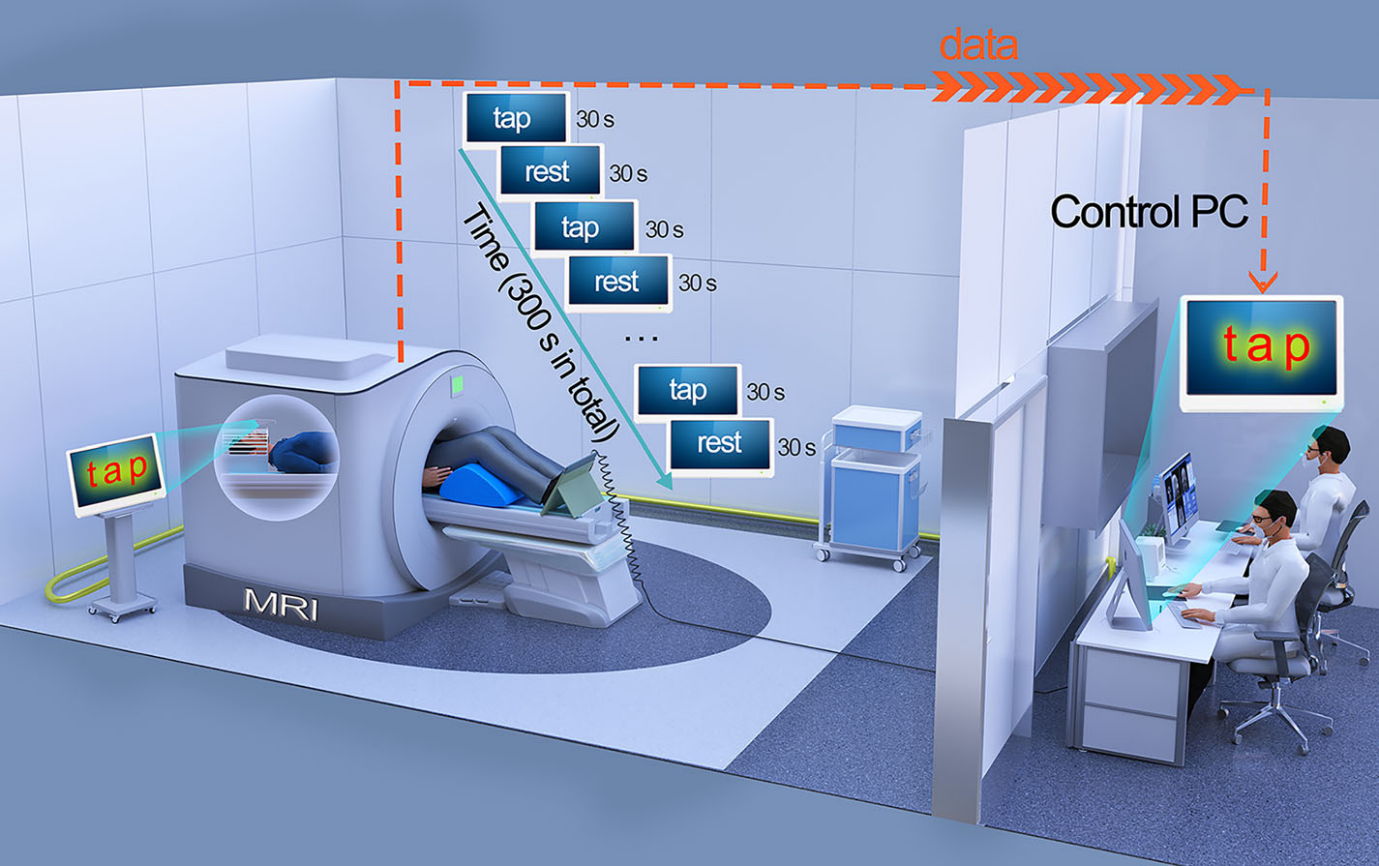
Schematic diagram of the fMRI assessment. Participants were instructed to flex and extend their ankles at a preferred rhythm for 30 s when they saw “tap” and rest with their eyes open for 30 s when they saw “rest.” The counts of footsteps were recorded.

For the foot tapping task, we used a block design in which the task period (30 s) consisted of dorsiflexion and plantarflexion (simulating walking posture), and the resting period (30 s) consisted of relaxing with eyes open. The entire task lasted for a total time of 300 s (Figure 1). Subjects lay supine inside the MRI scanner with knee flexion of about 30° and feet positioned on the pedals (Figure 1). A mirror was mounted to the head coil to ensure that subjects would have a clear view of instructions projected on the screen. Participants were required to tap the pedals according to visual stimuli (“tap”). After 30 s of tapping the pedals, participants were required to rest according to the visual stimuli (“rest”) projected onto the mirror for 30 s. The pedals were linked to a computer by a universal serial bus connection, and all subject responses were thusly recorded. Before scanning, we explained the whole procedure in detail to the subjects and gave them practice exercises to ensure that they understood the test.

#### 2.4.2 fMRI Acquisition and Preprocessing

MRI scans were obtained using a General Electric signal 3.0 T magnetic resonance scanner (Pioneer, General Electric Company, Boston, MA, USA) during the ON state (to minimize head motion) before and after rehabilitation. The head coil was filled with foam to limit head movements, and earplugs were used to reduce noise. fMRI data were obtained using a single‐shot gradient‐echo echo‐planar imaging (EPI) sequence with the following parameters: field of view (FOV) = 280 × 280 mm^2^, 40 slices per volume, interleaved slice order, voxel size 2.2 × 2.2 × 4 mm^3^, acquisition matrix 300 × 300, flip angle = 90°, echo time (TE) = 30 ms, repetition time (TR) = 2 s, and acquisition time (TA) = 2 s. To ensure signal stability, we set 5 EPI scans before the start of the task, therefore, 155 EPI volumes were obtained in total for each patient. In addition, a high‐resolution 3D T1‐weighted sagittal scan was acquired for each subject (voxel size 1 × 1 × 1 mm^3^ isotropic; acquisition matrix 300 × 300 × 164).

Preprocessing of functional scans was performed using Statistical Parametric Mapping Software (SPM12; Wellcome Department of Cognitive Neurology, London, UK), and the first five volumes of the functional images were discarded. The preprocessing of functional scans proceeded as follows: (1) slice time was corrected to the median (20th) slice in each repetition time; (2) motion correction was carried out (six parameter affine transformation, realignment to the mean image, and mean created using 4th spline interpolation); (3) co‐registration of the T1 image to the mean echo planar imaging (normalized mutual information) was conducted, as well as co‐registration of these images to the SPM average T1 image; (4) normalization was performed for East Asian brains according to the international consortium for brain mapping space template to reduce morphing errors (Zhang et al. 2017); and (5) images were smoothed using an 8‐mm full‐width at half‐maximum isotropic Gaussian kernel.

#### 2.4.3 Task Activation Analysis

The neural responses to “tap” in the priming task were examined with a two‐level general linear model (GLM). At the first level, we modeled the “tap” stage as a regressor and the “rest” stage as an implicit baseline, and the regressor was convolved with the canonical hemodynamic response function. Six head movement parameters provided by motion correction were later modeled in the GLM to control for the possible effects of head motion. Finally, a contrast (compared to the “rest” stage) was constructed to investigate brain responses during the task stage (Xu et al. 2022). Once the GLM model was estimated, a regression coefficient matrix was obtained to evaluate the brain activation (“tap” stage > “rest” stage) for each subject. At the second level, the “combined” images from each group were analyzed by one‐sample *t*‐test to obtain the group‐level activation. After this, two‐sample *t*‐tests were carried out to test the differences in activations between groups (HCs vs. pre‐NTD‐PD, HCs vs. pre‐TD‐PD, pre‐NTD‐PD vs. pre‐TD‐PD, pre‐NTD‐PD vs. post‐NTD‐PD, and pre‐TD‐PD vs. post‐TD‐PD). Hoehn–Yahr stage and LEDs were included as covariates when comparing the difference in activations between the pre‐NTD‐PD and pre‐TD‐PD groups.

#### 2.4.4 Connectivity Analysis

To explore the effect of rehabilitation‐induced alterations on FC in TD‐PD and NTD‐PD patients further, we conducted FC analysis using the Graph Theory Network Analysis toolbox (Wang et al. 2015). First, the brain was parcellated into 116 regions of interest (ROIs) using the automated anatomical labeling brain template (Tzourio‐Mazoyer et al. 2002). After that, we calculated the mean of the fMRI time series of all voxels in each ROI. To obtain a symmetric FC matrix, the Pearson correlation coefficient between any pair of 116 ROIs was calculated. To improve the normality of the correlation coefficient, the Fisher *r*‐to‐*z* transformation was used to transform the correlation matrix into a *z*‐score symmetric matrix. Then, two‐sample *t*‐tests were carried out to test the differences in FC between groups. We compared the FC differences between NTD‐PD patients, TD‐PD patients, and HCs at baseline. As above, Hoehn–Yahr stage and LEDs were included as covariates when comparing the FC difference between pre‐NTD‐PD and pre‐TD‐PD groups. We also compared the differences in FC before and after rehabilitation in NTD‐PD and TD‐PD patients as well.

All the evaluations and treatments were performed in the ON condition (under regular dopaminergic medication), and the same assessors performed evaluations at baseline and after rehabilitation. This study was approved by the Institutional Ethics Committee of Beijing Rehabilitation Hospital, Capital Medical University of China (approval No. 2018bkky022) on May 7, 2018, and all procedures performed were in accordance with the 1964 Helsinki Declaration. Informed consents were obtained from all participants before inclusion.

### 2.5 Statistical Analysis

#### 2.5.1 Clinical Measurements

The Shapiro–Wilk test was used to assess the distribution of the data. The mean ± standard deviation was reported for normally distributed data, and the median (interquartile range) was reported for non‐normal data. Comparisons of sociodemographic variables between the HCs, TD, and NTD groups at baseline were performed using one‐way ANOVA for continuous variables and the Chi‐square test for binary variables. The two‐sample *t*‐test was used to analyze the differences in basic clinical information between the TD group and the NTD group for normally distributed data and the Mann–Whitney test for non‐normal data. Changes in motor functional variables after rehabilitation in the TD group and the NTD group were analyzed using the paired‐samples *t*‐test or the Wilcoxon signed‐rank test depending on the distribution of the data. Effect size was calculated using Cohen's *d* and matched rank biserial correlation for Student's *t*‐test and Wilcoxon test, respectively. To account for multiple comparisons, two‐sided *p* values were adjusted according to the method of Benjamini/Hochberg (B/H) to control the false discovery rate (FDR). A corresponding B/H‐adjusted *p* value below 0.05 was considered to be statistically significant. All analysis was performed using JASP (Version 0.16.2).

#### 2.5.2 fMRI Measurements

For the brain activation analysis, the results of within‐group or between‐group comparisons were reported at *p* < 0.001, and only clusters > 5 voxels were considered (Sarasso et al. 2021). For the FC analysis, *p* < 0.001 was used as the threshold for statistical significance of two‐sample *t*‐tests given the relatively small sample (Zhang et al. 2017). For exploratory purposes, all findings were shown at *p* < 0.001 uncorrected, as the present study aims to investigate any possible effects of rehabilitation on brain functions (Bender and Lange 2001).

#### 2.5.3 Brain‐Behavior Correlation Analysis

To evaluate and graph the linear correlation between clinical assessments and FC values, we calculated the Pearson correlation between changes in FC and changes in clinical measurements after rehabilitation.

## 3 Results

### 3.1 Baseline Clinical Characteristics

Of 69 PWPs, 41 PWPs had complete fMRI data before and after rehabilitation. Four PWPs in the NTD group and two PWPs in the TD group were withdrawn due to head movements exceeding 6 mm or rotations greater than 6°. In total, there were 24 PWPs in the NTD group and 11 PWPs in the TD group. The clinical characteristics and counts of footsteps during the fMRI task were comparable between groups (Table 1).

**TABLE 1:** Demographic profiles of the study sample.

	HCs (*n* = 21)	NTD group (*n* = 24)	TD group (*n* = 11)	*p* value^a^	*p* value^b^
Sex [male, *n* (%)]	10 (47.6)	15 (62.5)	7 (63.6)	1.000	—
Age, (years)	60 ± 9	60 ± 8	57 ± 8	0.904	—
Height (m)	1.64 ± 0.09	1.67 ± 0.09	1.68 ± 0.06	0.893	—
Weight (kg)	65.58 ± 9.26	64.73 ± 8.56	69.73 ± 6.31	0.746	—
Counts of footsteps (/30 s)	38 ± 11	35 ± 17	36 ± 12	0.747	
Disease duration, years	—	5.9 ± 2.7	5.7 ± 2.7	—	0.996
LEDs (mg)	—	653.26 ± 237.78	437.50 ± 110.11	—	0.074
Hoehn–Yahr stage	—	2.8 (1.0)	2 (1.0)	—	0.076
MMSE	—	28 (2)	29 (3)	—	0.17
MDS‐UPDRS III	—	29 ± 10	27 ± 11	—	0.826
BBS	—	54 (3)	55 (2)	—	0.996
TUG (s)	—	9.45 ± 1.50	9.19 ± 1.25	—	0.996
10MWT‐Comfortable gait speed (m/s)	—	1.12 ± 0.16	1.24 ± 0.16	—	0.424
10MWT‐Fast gait speed (m/s)	—	1.55 ± 0.22	1.55 ± 0.15	—	0.999
6MWT (m)	—	480 (115)	479 (84)	—	0.826

*Note*: Normally distributed data were expressed as the mean ± standard deviation, and non‐normal data were reported as the median (interquartile range).

Abbreviations: 10MWT, 10‐Meter Walk Test; 6MWT, 6‐Minute Walk Test; BBS, Berg Balance Scale; HCs, healthy controls; LEDs, levodopa equivalent doses; MMSE, Mini‐Mental State Examination; NTD, non‐tremor dominant; PD, Parkinson's disease; TD, tremor dominant; TUG, the Timed up and Go test; MDS‐UPDRS III, Movement Disorder Society United Parkinson's Disease Rating Scale Part III.

^a^ *p*, comparisons between HCs, the NTD group, and the TD group.

^b^ *p*, comparisons between the NTD group and the TD group.

### 3.2 Changes in Motor Functional Variables After Rehabilitation

The performance of MDS‐UPDRS III, TUG, 10MWT, and 6MWT were improved in the NTD group and the TD group after 2‐week rehabilitation (Tables 2 and 3). In addition, there were significant changes in axial subscore, bradykinesia subscore, BBS, and counts of footsteps in the NTD group (Table 2).

**TABLE 2:** Changes in motor function after rehabilitation in NTD group.

	Pre‐MIRT	Post‐MIRT	*p* value	Effect size
MDS‐UPDRS III	28 (7)	24 (13)	< 0.001	0.981
Axial subscore	5 (1)	4 (1)	0.002	1.000
Bradykinesia subscore	14 (7)	10 (6)	0.002	0.926
Tremor subscore	3 (4)	3 (4)	0.064	0.821
Rigidity subscore	7 (7)	6 (5)	0.222	0.500
BBS	54 (3)	55 (2)	0.004	−1.000
TUG (s)	9.15 (1.88)	8.27 (1.41)	< 0.001	1.000
10MWT‐Comfortable gait speed (m/s)	1.12 (0.17)	1.23 (0.19)	0.001	−0.873
10MWT‐Fast gait speed (m/s)	1.55 ± 0.22	1.65 ± 0.23	0.006^a^	−0.642
6MWT (m)	453 ± 87	500 ± 72	< 0.001^a^	−1.155
Counts of footsteps (/30 s)	35 ± 17	40 ± 16	0.002^a^	−0.760

*Note*: Normally distributed data were expressed as the mean ± standard deviation, and non‐normal data were reported as the median (interquartile range). For the Student *t*‐test, effect size is given by Cohen's *d*. For the Wilcoxon test, effect size is given by the matched rank biserial correlation.

Abbreviations: 10MWT, 10‐Meter Walk Test; 6MWT, 6‐Minute Walk Test; BBS, Berg Balance Scale; MDS‐UPDRS III, Movement Disorder Society United Parkinson's Disease Rating Scale Part III; NTD, non‐tremor dominant; TUG, Timed up and Go test.

^a^ *p* values were obtained using paired‐samples *t*‐test; the rest were Wilcoxon signed‐rank test.

**TABLE 3:** Changes in motor function after rehabilitation in TD group.

	Pre‐MIRT	Post‐MIRT	*p* value	Effect size
MDS‐UPDRS III	27 ± 11	23 ± 11	0.023^a^	0.944
Axial subscore	3 (3)	2 (2)	0.122	1.000
Bradykinesia subscore	11 ± 5	10 ±5	0.128^a^	0.584
Tremor subscore	7 (4)	7 (3)	0.191	1.000
Rigidity subscore	5 (4)	3 (6)	0.116	1.000
BBS	55 (2)	55 (2)	0.098	−1.000
TUG (s)	9.19 ± 1.25	8.18 ± 1.29	0.006^a^	1.492
10MWT‐Comfortable gait speed (m/s)	1.24 ± 0.16	1.35 ± 0.17	0.025^a^	−0.972
10MWT‐Fast gait speed (m/s)	1.56 (0.21)	1.69 (0.18)	0.022	−1.000
6MWT (m)	479 (84)	522 (51)	0.005	−1.000
Counts of footsteps (/30 s)	36 ± 12	39 ± 12	0.174	−0.442

*Note*: Normally distributed data were expressed as the mean ± standard deviation, and non‐normal data were reported as the median (interquartile range). For the Student *t*‐test, effect size is given by Cohen's *d*. For the Wilcoxon test, effect size is given by the matched rank biserial correlation.

Abbreviations: 10MWT, 10‐Meter Walk Test; 6MWT, 6‐Minute Walk Test; BBS, Berg Balance Scale; MDS‐UPDRS III, Movement Disorder Society United Parkinson's Disease Rating Scale Part III; TD, tremor‐dominant; TUG, the Timed up and Go test.

^a^ *p* values were obtained using paired‐samples *t*‐test; the rest were Wilcoxon signed‐rank test.

### 3.3 Baseline Brain Activation Within and Between Groups

During the fMRI task, HCs showed bilateral activation of the paracentral lobule (Figure S1A–C; Table S1). NTD‐PD patients showed recruitment of the right supplementary motor area (SMA), the left postcentral gyrus, the left cerebellum, and both supramarginal gyri (Figure S1B; Table S1). TD‐PD patients activated both inferior frontal gyri (IFG), the left Rolandic operculum, and the right insula (Figure S1C; Table S1). Compared to the HCs, NTD‐PD patients showed increased activation in the left postcentral gyrus (Figure S1D; Table S1), and TD‐PD patients showed increased activation in the left IFG, and the right insula (Figure S1E; Table S1). However, there was no difference in the brain activation between NTD‐PD and TD‐PD patients after adjusting for Hoehn–Yahr stage and LEDs.

### 3.4 Baseline FC Between Groups

Compared to the HCs, the NTD‐PD patients showed increased FC between the left IFG and right supramarginal gyri, the right anterior cingulate gyrus and right supramarginal gyrus, and decreased FC between the cerebellum and the precental gyrus, the temporal lobe, and the occipital lobe, as well as within the cerebellar (*p* < 0.001 uncorrected; Figure S2; Table S2). TD‐PD patients showed increased FC between the left precental gyrus and right IFG, and the frontal lobe and the cerebellum compared to the HCs (Figure S3; Table S2). Decreased FC was also found in TD‐PD patients relative to HCs (Figure S3; Table S2). Compared to TD‐PD patients, NTD‐PD patients showed increased FC between the cerebellum and the frontal, the temporal, and the occipital lobes (Figure S4C; Table S2).

### 3.5 Changes in Brain Activations in the NTD and TD Groups Pre‐ and Postrehabilitation

**FIGURE 2:**
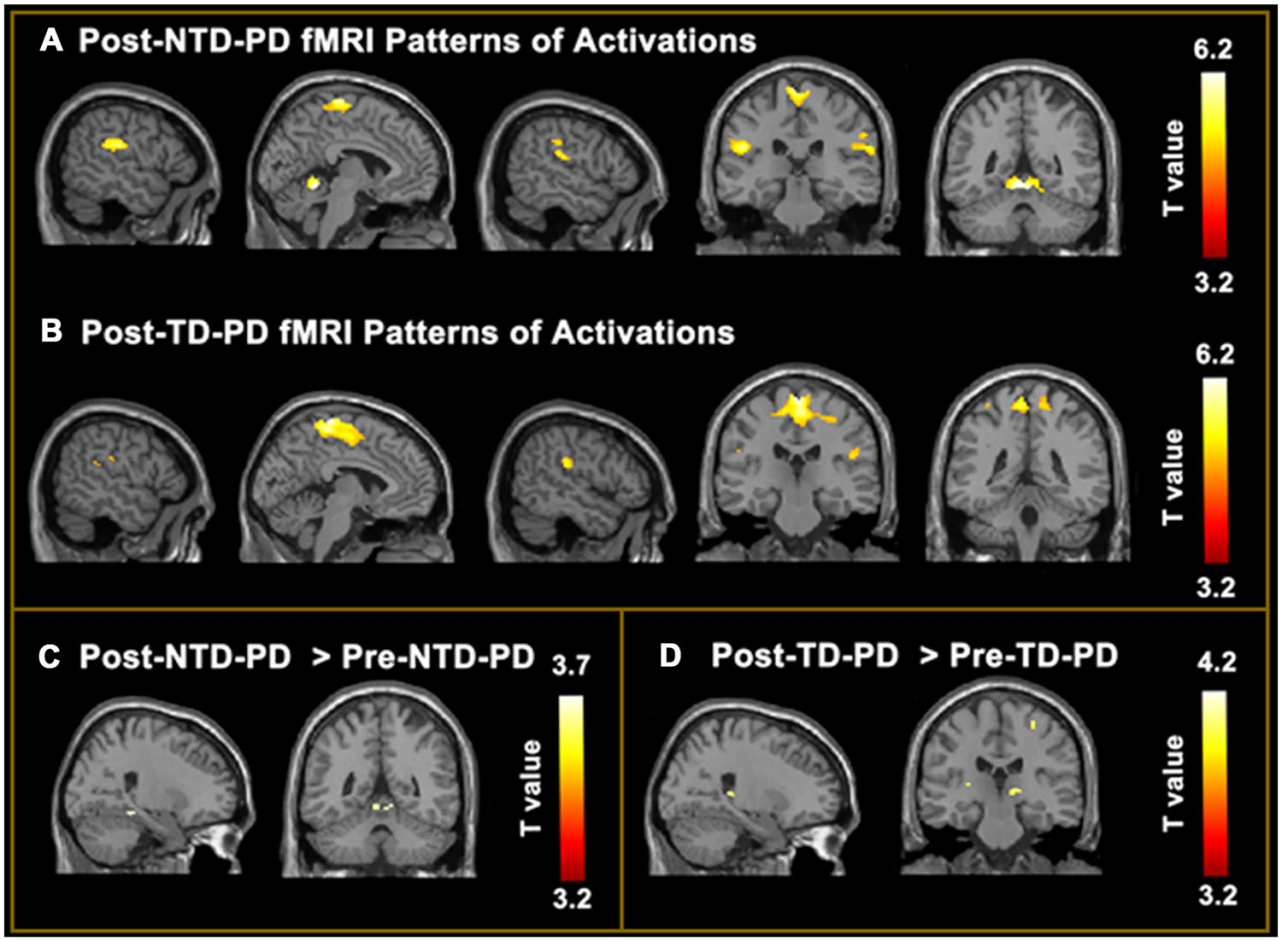
fMRI patterns of changes after rehabilitation in the NTD and TD groups. (A) Brain activity during the foot tapping task after rehabilitation in NTD‐PD patients and (B) TD‐PD patients. (C) Changes in brain activity after rehabilitation relative to baseline in NTD‐PD patients and (D) TD‐PD patients. All results are shown at *p* < 0.001 uncorrected, and only clusters > 5 voxels are reported. Color bars denote *t* values. NTD, non‐tremor dominant; TD, tremor dominant.

Brain activations in the NTD group and TD group during the foot tapping task after rehabilitation were shown in Figure 2A,B. After 2‐week rehabilitation, the NTD group showed increased recruitments of the right parahippocampal gyrus, vermis IV and V, left cerebellum, and right lingual gyrus (Figure 2C; Table S3). The TD group showed increased activations of the left postcentral gyrus, bilateral thalamus, right hippocampus, right precental gyrus, and right insula (Figure 2D; Table S3).

### 3.6 Alterations in FC in the NTD and TD Groups Pre‐ and Postrehabilitation

**FIGURE 3:**
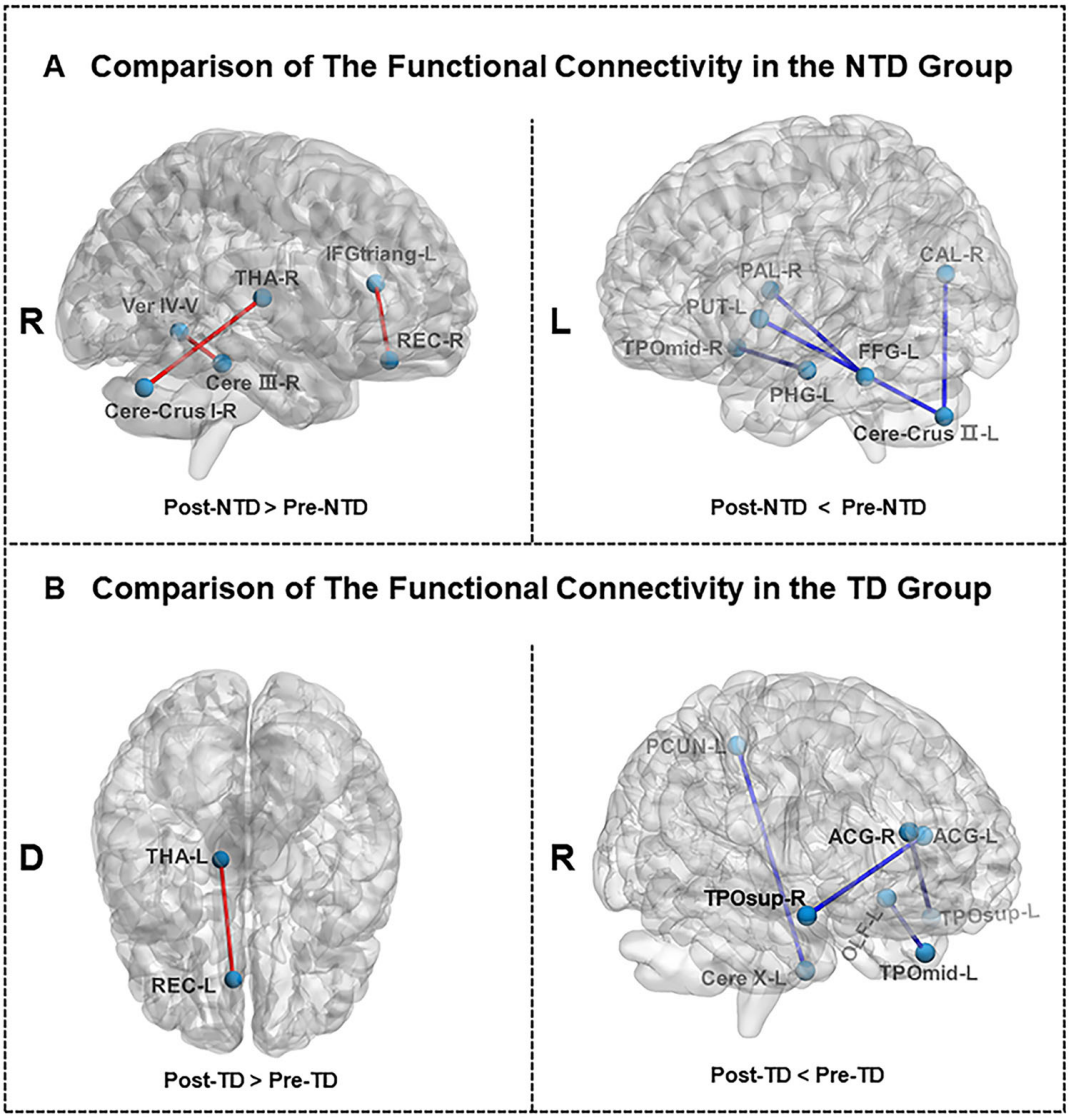
Changes in FC of PWPs after rehabilitation. (A) Alterations in FC of NTD‐PD patients after rehabilitation. (B) Changes in FC of TD‐PD patients after rehabilitation. The heavy line refers to areas on the surface of the brain, and the light line refers to the areas in the interior of the brain. All results are shown at *p* < 0.001 uncorrected. ACG, anterior cingulate and paracingulate gyri; CAL, calcarine fissure and surrounding cortex; Cere, cerebellum; D, down view; FFG, fusiform gyrus; IFGtriang, triangular part of the inferior frontal gyrus; L, left hemisphere; OLF, olfactory cortex; PAL, pallidum; PCUN, precuneus; PHG, parahippocampal gyrus; PUT, putamen; R, right hemisphere; REC, gyrus rectus; THA, thalamus; TPOmid, middle temporal gyrus of the temporal pole; TPOsup, superior temporal gyrus of the temporal pole; Ver, vermis.

As shown in Figure 3A, NTD‐PD patients showed increased FC between the left IFG and right gyrus rectus, the right thalamus and right cerebellum crus I, and the right cerebellum lobule III and vermis IV and V after rehabilitation. Reduced FC between the left fusiform gyrus and right pallidum and the left putamen and left cerebellum crus II were also observed in NTD‐PD patients (Figure 3A; Table S4). TD‐PD patients showed increased FC between the left gyrus rectus and left thalamus and decreased FC between the anterior cingulate gyri and temporal gyrus of the temporal pole, and the left precuneus and left cerebellum after treatment (Figure 3B; Table S4).

### 3.7 Correlations Between Alterations in FC and Clinical Changes

**FIGURE 4:**
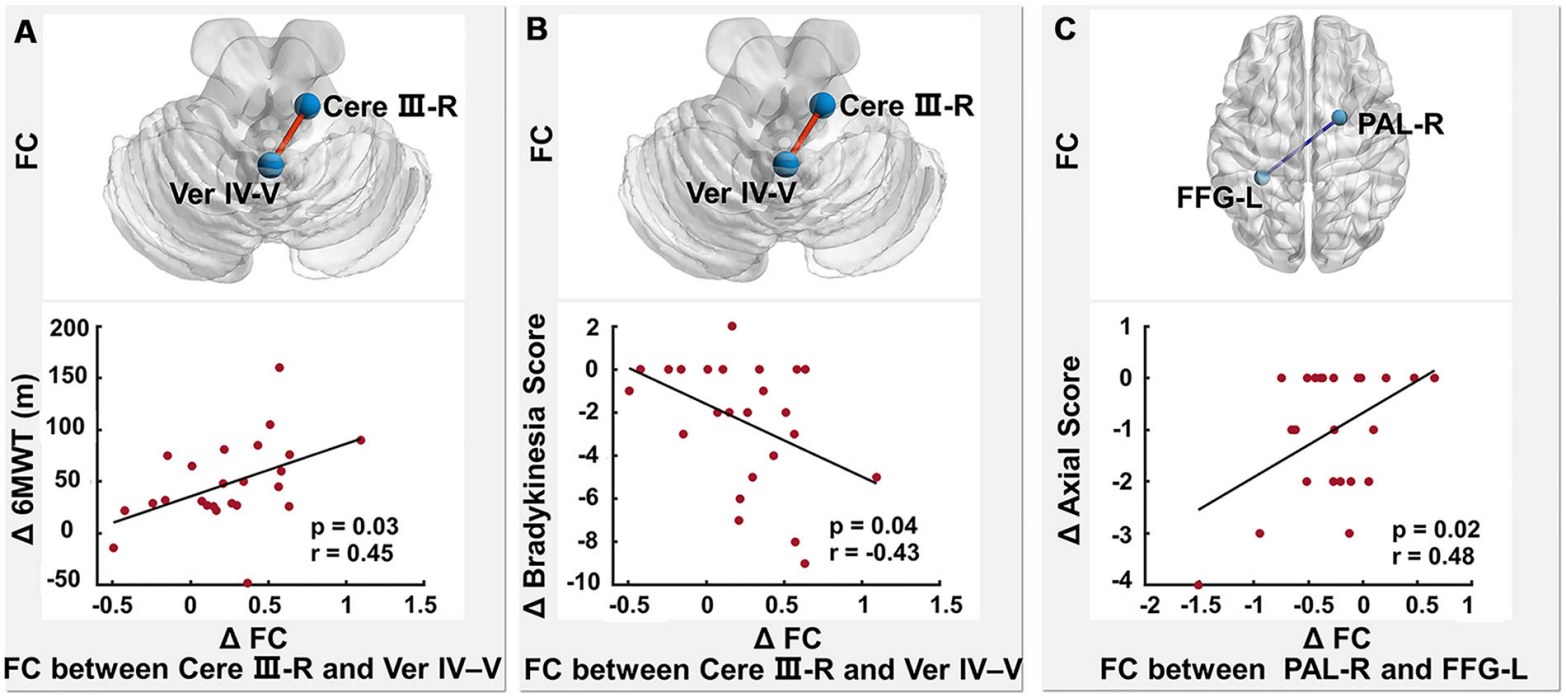
Changes in FC after rehabilitation relative to baseline correlated with clinical changes in the NTD group. (A) Increased FC between the right cerebellum lobule III and vermis IV and V correlated positively with increased 6MWT after rehabilitation relative to baseline. (B) Increased FC between right cerebellum lobule III and vermis IV and V was associated with reduced bradykinesia subscore after rehabilitation relative to baseline. (C) Decreased FC between the left fusiform gyrus and right pallidum correlated positively with reduced axial subscore after rehabilitation relative to baseline. ∆, changes in measurements after rehabilitation (post‐rehabilitation–pre‐rehabilitation); 6MWT, 6‐Minute Walk Test; Cere, cerebellum; FFG, fusiform gyrus; PAL, pallidum; Ver, vermis.

In the NTD group, increased FC between the right cerebellum lobule III and vermis IV and V correlated positively with increased 6MWT (*r* = 0.45, *p* = 0.03; Figure 4A) and reduced bradykinesia subscore (*r* = −0.43, *p* = 0.04; Figure 4B), and decreased FC between the left fusiform gyrus and right pallidum correlated positively with reduced axial subscore (*r* = 0.48, *p* = 0.02; Figure 4C). There were no significant correlations between changes in FC and motor symptoms in the TD group.

## 4 Discussion

In this study, we examined changes in motor performance, brain activation, and FC related to 2‐week rehabilitation in TD‐PD and NTD‐PD patients, respectively. Our results indicate that rehabilitation improved motor functions in PWPs regardless of motor subtype. However, rehabilitation‐induced brain function changes have distinct characteristics in NTD‐PD patients and TD‐PD patients. Specifically, NTD‐PD patients showed higher activation in the cerebellum and increased within‐cerebellar connectivity after rehabilitation, while TD‐PD patients showed increased recruitments of the sensorimotor cortex and the bilateral thalamus.

Clinical findings suggest that both groups showed improvements of motor functions after rehabilitation, and these results are congruent with the existing literature (Chen et al. 2021; Ferrazzoli et al. 2018). This suggests that rehabilitation is effective in improving cardinal motor symptoms, balance, and gait in PWPs regardless of motor subtype.

We found that both HC and PWPs showed activation of the paracentral lobules during the foot tapping task. NTD‐PD patients also showed additional activations of the SMA, the postcentral gyrus, the supramarginal gyrus, and the cerebellum. The SMA is essential to motor planning and initiation (Tanji 1994), and excessive inhibition of the basal ganglia to the thalamus leads to hypoactivation of the SMA, which is related to akinesia, bradykinesia, and gait disorders in PWPs (Grafton 2004). fMRI tasks may alter the activation of SMA in PWPs (Xing et al. 2020). PWPs exhibited more activity in the SMA during complex walking compared to usual walking (Peterson et al. 2014). In addition, the SMA plays an important role in human postural control (Dijkstra et al. 2020). The SMA has been found to be a hub in patients with freezing of gait when an anticipatory postural adjustment is required for a task (de Lima‐Pardini et al. 2020). NTD‐PD patients might therefore require more anticipatory postural adjustments for step initiation, which activates the SMA.

Located in the ventral parietal cortex, the supramarginal gyrus, together with the ventral frontal regions, is associated with the attention network (Corbetta and Shulman 2002). PWPs show increased use of executive‐attentional resources during real‐time walking and balance tasks to compensate for gait and balance deficits (Stuart et al. 2019; Stuart et al. 2018). Recent research has also reported that NTD‐PD patients recruit more cognitive resources for walking compared to TD‐PD patients (Orcioli‐Silva et al. 2021). Thus, recruitment of the supramarginal gyrus and increased FC between the IFG and the supramarginal gyrus during the foot tapping task in the NTD‐PD group might indicate that NTD‐PD patients need additional recruitment of cognitive resources to compensate for movement automaticity deficits.

The cerebellum is a key node of human postural control (Dijkstra et al. 2020) and is also involved in the regulation of balance and gait. PD‐PIGD patients have increased activity of the cerebellum relative to HCs when performing automatic movements (Sarasso et al. 2021), suggesting that hyperactivation of the cerebellum is a compensatory mechanism for defective basal ganglia (Yu et al. 2007). In this study, we also found that NTD‐PD patients compared to HCs showed reduced within‐cerebellar connectivity and decreased FC between the cerebellum and the precental gyrus, the temporal lobe, and the occipital lobe, which is in line with previous research (Festini et al. 2015). Therefore, we speculate that the cerebellar activation task in NTD‐PD patients during the foot tapping task may be a compensatory effect for inefficient communication within the cerebellum and between the cerebellum and other brain regions or for deficits of the basal ganglia.

Compared to HCs, TD‐PD patients showed higher activations in the left IFG and right insula during the foot tapping task. The left IFG is responsible for monitoring relevant and irrelevant actions as well as selecting appropriate actions (Milham et al. 2001; Swick, Ashley, and Turken 2008). Activation of the IFG in the TD‐PD group may be due to the presence of resting tremors, which are irrelevant actions for foot tapping. Although there were no differences in brain activations between NTD‐PD and TD‐PD patients, NTD‐PD patients showed greater FC of the cerebellum with the frontal, temporal, and occipital cortices, which suggests that these two motor subtypes have distinct brain network patterns.

Concerning rehabilitation‐induced changes in fMRI, the NTD group showed increased activity in the vermis and left cerebellum lobules IV and V. Wu and Hallett (2013) have indicated that the compensatory effect of the cerebellum may help maintain better motor and nonmotor functions in PWPs, and studies have also suggested that rehabilitation‐induced cerebellar hyperactivity is correlated with better balance and restoration of gait automation in PWPs (Sarasso et al. 2021; Vieira‐Yano et al. 2021). Indeed, we found increased within‐cerebellar connectivity after rehabilitation in the NTD group.

Increased within‐cerebellar connectivity in PWPs is closely associated with motor or cognitive function (Mirdamadi 2016). PWPs off medication compared to HCs showed increased within‐cerebellar connectivity that related to improved cognitive and motor performance (Festini et al. 2015). In this study, greater within‐cerebellar connectivity correlated positively with enhanced 6MWT and reduced bradykinesia subscore, suggesting that strengthening the within‐cerebellar connectivity may improve the motor symptoms of NTD‐PD patients. Although counts of footsteps during the foot tapping task were significantly improved in the NTD group after rehabilitation, we found no significant direct or indirect associations between changes in the counts of footsteps and brain alterations in the NTD group. We speculate that counts of footsteps, a simple indicator, cannot fully reflect the changes in local brain function induced by rehabilitation but merely reflect that the subjects performed the test according to the experimental requirements.

Right cerebellar crus I is associated with cognitive function (Guell and Schmahmann 2020), and a recent study using positron emission tomography found a positive correlation between the metabolism of the right cerebellar crus I and the metabolism of the bilateral thalamus in PWPs and also found that metabolically increased cerebello‐thalamic loops were related to poor executive performance (Riou et al. 2021). Thus, increased FC between the right thalamus and right cerebellar crus I after rehabilitation in the NTD group might be associated with changes of cognitive functions such as attention ability, which warrants further study.

In the TD group, fMRI showed different patterns of functional changes after rehabilitation. These patients showed increased activations of motor control and sensory integration areas after rehabilitation, including the sensorimotor cortex and both sides of the thalamus. Decreased UPDRS III has been found to be related to increased activation of the precentral gyrus during the foot movement task after rehabilitation (Agosta et al. 2017), suggesting that increased recruitment of the motor network after rehabilitation might be the underlying mechanism of motor improvement in TD‐PD patients. We also found an increased recruitment of the right hippocampus in the TD group after rehabilitation. In older adults, increased stride length variability and decreased stride length are related to lower activity and smaller volume of the hippocampus, respectively (Zimmerman et al. 2009).

The anterior cingulate cortex and the temporal pole are involved in cognitive function (Bush, Luu, and Posner 2000; Herlin, Navarro, and Dupont 2021), and alterations in FC in the TD group after rehabilitation may be related to cognitive function. However, cognitive assessments were not performed in this study because the training time was short and did not exceed the washout period for cognitive assessments. The relationship between FC changes and cognitive changes in TD‐PD patients after rehabilitation warrants further study.

To the best of our knowledge, this is the first study to investigate the rehabilitation‐induced alterations of neural activation and FC in TD‐PD patients and NTD‐PD patients. There are several limitations to the study, however. First, the sample size is relatively small, but it is important to consider the difficulty of recruiting a sample of PD patients able to perform fMRI before and after rehabilitation. Second, PWPs were evaluated in the “on” state, which may not reflect their true disease state. Third, this is an exploratory study, and fMRI results were not corrected for multiple comparisons. Therefore, fMRI results should be interpreted carefully. Fourth, there is still controversy about the motor subtyping of PD. Some studies have indicated that the TD subtype is the early stage of PD and eventually shifts to the NTD subtype with disease progression (Simuni et al. 2016; von Coelln et al. 2021). However, motor subtyping is still the most commonly used clinical subtyping method, and numbers of studies have concluded that clinical symptoms, disease progression, and therapeutic efficacy of the two subtypes are different. Data‐driven subtyping systems may be more valuable for disease prognostic research in the future.

In conclusion, we found different FC of the brain during the foot tapping task in the NTD group compared to the TD group. In addition, rehabilitation‐induced brain functional reorganization varied by motor subtypes. After rehabilitation, compensatory effects of the cerebellum increased in the NTD group, whereas activations of motor control and sensory integration areas were enhanced in the TD group.

## Author Contributions

Conception and study design: Boyan Fang and Chunlin Li. Data collection or acquisition: Zhaohui Jin, Yixuan Wang, Detao Meng, Xin Yu, Mengyue Wang, Meng Lin, Qiping Wen, and Keke Chen. Statistical analysis: Keke Chen and Songjian Wang. Interpretation of results: Boyan Fang, Chunlin Li, and Youwei Li. Drafting the manuscript work: Keke Chen and Songjian Wang. Revising the manuscript critically for important intellectual content: Boyan Fang and Chunlin Li. Approval of final version to be published and agreement to be accountable for the integrity and accuracy of all aspects of the work: Keke Chen, Songjian Wang, Qiping Wen, Zhaohui Jin, Yixuan Wang, Detao Meng, Xin Yu, Mengyue Wang, Meng Lin, Youwei Li, Chunlin Li, and Boyan Fang.

## Disclosure

The authors have nothing to report.

## Ethics Statement

All procedures performed in studies involving human participants were in accordance with the ethical standards of the institutional and/or national research committee and with the 1964 Helsinki Declaration and its later amendments or comparable ethical standards. This study was approved by the Institutional Ethics Committee of Beijing Rehabilitation Hospital, Capital Medical University of China (approval No. 2018bkky022) on May 7, 2018.

## Consent

Informed consents were obtained from all participants before inclusion.

## Conflicts of Interest

The authors declare no conflicts of interest.

### Peer Review

The peer review history for this article is available at https://publons.com/publon/10.1002/brb3.70102

## Supporting information



Supporting Information

Supporting Information

Supporting Information

Supporting Information

Supporting Information

## Data Availability

The data that support the findings of this study are available from the corresponding author upon reasonable request.
