# Supplementary material for: Rehabilitation Response in Tremor‐ and Non‐Tremor‐Dominant Parkinson Disease: A Task‐fMRI Study
Source: Brain Behav. 2024 Oct 17;14(10):e70102. doi: 10.1002/brb3.70102 (PMC11483598; doi:10.1002/brb3.70102)
Supplement: Supplementary file 1 — Supporting Information [file BRB3-14-e70102-s004.docx]

**Supplementary Table 1. Baseline fMRI patterns of activation during the foot tapping task**

| **HCs** | | | | | | | |
| --- | --- | --- | --- | --- | --- | --- | --- |
| ***KE*** | ***p*** | ***p*_FWE_** | **T** | **mni x** | **mni y** | **mni z** | **Area** |
| 219 | < 0.001 | 0.076 | 4.37 | 6 | -26 | 68 | Paracentral_Lobule_R |
|  |  |  | 3.23 | -2 | -30 | 64 | Paracentral_Lobule_L |
| **NTD group** | | | | | | | |
| 1220 | < 0.001 | < 0.001 | 7.71 | 2 | -24 | 70 | Paracentral_Lobule_R |
|  |  |  | 6.64 | 2 | -12 | 68 | Supp_Motor_Area_R |
| 236 | < 0.001 | 0.065 | 4.42 | -54 | -24 | 22 | SupraMarginal_L |
|  |  | 0.169 | 4.09 | -58 | -16 | 18 | Postcentral_L |
| 68 | < 0.001 | 0.323 | 3.83 | 60 | -24 | 20 | SupraMarginal_R |
| 9 | < 0.001 | 0.421 | 3.71 | -4 | -50 | -12 | Cerebelum IV–V_L |
| **TD group** | | | | | | | |
| 111 | < 0.001 | 0.006 | 5.14 | -2 | -24 | 72 | Paracentral_Lobule_L |
| 7 | < 0.001 | 0.688 | 3.41 | -40 | 20 | 12 | Frontal_Inf_Tri_L |
| 14 | < 0.001 | 0.695 | 3.41 | -50 | -22 | 22 | Rolandic_Oper_L |
| 5 | < 0.001 | 0.718 | 3.38 | 52 | 10 | 12 | Frontal_Inf_Oper_R |
| 8 | < 0.001 | 0.780 | 3.31 | 38 | 6 | 16 | Insula_R |
| **NTD group > HCs** | | | | | | | |
| 14 | < 0.001 | 0.595 | 3.52 | -58 | -14 | 16 | Postcentral_L |
| **TD group > HCs** | | | | | | | |
| 13 | < 0.001 | 0.608 | 3.50 | -40 | 20 | 12 | Frontal_Inf_Tri_L |
| 17 | < 0.001 | 0.745 | 3.35 | 34 | 4 | 18 | Insula_R |

**Supplementary Table 2. Baseline functional connectivity between groups during the foot tapping task**

| **NTD group > HCs** | | | |
| --- | --- | --- | --- |
| Regions | Regions | T value | P value |
| Frontal_Inf_Tri_L | SupraMarginal_R | 3.63 | < 0.001 |
| Supp_Motor_Area_L | Pallidum_L | 3.70 | < 0.001 |
| Cingulum_Ant_R | SupraMarginal_R | 3.54 | < 0.001 |
| Parietal_Sup_L | Temporal_Sup_R | 3.57 | < 0.001 |
| Parietal_Sup_R | Pallidum_L | 5.01 | < 0.001 |
| **NTD group < HCs** | | | |
| Precentral_L | Paracentral_Lobule_R | - 3.96 | < 0.001 |
| Precentral_L | Cerebelum Crus Ⅱ_R | - 3.54 | < 0.001 |
| Frontal_Sup_L | Frontal_Sup_R | - 3.84 | < 0.001 |
| Supp_Motor_Area_R | Paracentral_Lobule_L | - 3.76 | < 0.001 |
| Rectus_R | Lingual_L | - 3.59 | < 0.001 |
| Insula_L | Cingulum_Ant_L | - 3.66 | < 0.001 |
| Insula_R | Lingual_L | - 3.57 | < 0.001 |
| Insula_R | Pallidum_L | - 4.43 | < 0.001 |
| Cingulum_Ant_R | Occipital_Inf_R | - 3.81 | < 0.001 |
| Hippocampus_L | Lingual_L | - 3.60 | < 0.001 |
| Calcarine_R | Vermis IV–V | - 4.02 | < 0.001 |
| Lingual_L | Lingual_R | - 3.93 | < 0.001 |
| Lingual_R | Vermis Ⅲ | - 4.59 | < 0.001 |
| Fusiform_L | Fusiform_R | - 3.83 | < 0.001 |
| Fusiform_L | Cerebelum IV–V_L | - 4.73 | < 0.001 |
| Fusiform_R | Temporal_Pole_Sup_L | - 5.01 | < 0.001 |
| Fusiform_R | Temporal_Pole_Sup_R | - 3.86 | < 0.001 |
| Putamen_R | Pallidum_L | - 3.57 | < 0.001 |
| Putamen_R | Thalamus_L | - 3.60 | < 0.001 |
| Putamen_R | Temporal_Sup_R | - 3.62 | < 0.001 |
| Pallidum_L | Temporal_Sup_R | - 4.31 | < 0.001 |
| Thalamus_R | Heschl_L | - 4.30 | < 0.001 |
| Thalamus_R | Temporal_Sup_L | - 4.26 | < 0.001 |
| Temporal_Sup_R | Temporal_Pole_Sup_R | - 4.18 | < 0.001 |
| Temporal_Sup_R | Cerebelum Ⅵ_L | - 3.63 | < 0.001 |
| Temporal_Inf_L | Cerebelum IV–V_R | - 4.36 | < 0.001 |
| Cerebelum Ⅲ_L | Vermis IV–V | - 3.73 | < 0.001 |
| Cerebelum Ⅲ_R | Vermis IV–V | - 3.76 | < 0.001 |
| Cerebelum IV–V_L | Cerebelum IV–V_R | - 4.52 | < 0.001 |
| **TD group > HCs** | | | |
| Precentral_L | Frontal_Inf_Orb_R | 4.81 | < 0.001 |
| Precentral_L | Cerebelum IV–V_R | 3.69 | < 0.001 |
| Precentral_R | Temporal_Pole_Sup_L | 3.74 | < 0.001 |
| Frontal_Mid_L | Cerebelum IV–V_R | 3.86 | < 0.001 |
| Cingulum_Post_R | Cerebelum Ⅹ_R | 3.96 | < 0.001 |
| Caudate_L | Pallidum_R | 4.61 | < 0.001 |
| **TD group < HCs** | | | |
| Precentral_L | Angular_L | - 5.03 | < 0.001 |
| Frontal_Sup_Orb_R | Heschl_L | - 4.32 | < 0.001 |
| Amygdala_R | Cerebelum Ⅵ_L | - 3.76 | < 0.001 |
| Fusiform_L | Fusiform_R | - 3.94 | < 0.001 |
| Fusiform_L | Temporal_Pole_Sup_L | - 4.53 | < 0.001 |
| Fusiform_R | Temporal_Pole_Sup_L | - 4.32 | < 0.001 |
| Fusiform_R | Temporal_Pole_Sup_R | - 3.87 | < 0.001 |
| **NTD group > TD group** | | | |
| Frontal_Mid_Orb_R | Cerebelum_Ⅹ_L | 4.10 | < 0.001 |
| Cuneus_L | Cerebelum_Ⅶb_R | 4.48 | < 0.001 |
| Fusiform_L | Putamen_L | 3.83 | < 0.001 |
| Fusiform_L | Vermis_Ⅵ | 3.99 | < 0.001 |

**Supplementary Table 3. fMRI patterns of activation after rehabilitation during the foot tapping task**

| **NTD group** | | | | | | | |
| --- | --- | --- | --- | --- | --- | --- | --- |
| ***KE*** | ***p*** | ***p*_FWE_** | **T** | **mni x** | **mni y** | **mni z** | **Area** |
| 323 | < 0.001 | 0.002 | 6.13 | -4 | -48 | -10 | Cerebelum IV–V_L |
|  | < 0.001 | 0.055 | 4.48 | 8 | -52 | -2 | Cerebelum IV–V_R |
|  | < 0.001 | 0.557 | 3.45 | 2 | -66 | 6 | Lingual_R |
| 516 | < 0.001 | 0.001 | 5.77 | 2 | -22 | 68 | Supp_Motor_Area_R |
|  | < 0.001 | 0.201 | 4.03 | 12 | -22 | 74 | Precentral_R |
| 365 | < 0.001 | 0.001 | 5.62 | -48 | -22 | 22 | Rolandic_Oper_L |
| 241 | < 0.001 | 0.039 | 4.59 | 62 | -26 | 22 | SupraMarginal_R |
| 15 | < 0.001 | 0.540 | 3.57 | 62 | -12 | 26 | Postcentral_R |
| **TD group** | | | | | | | |
| 2238 | < 0.001 | < 0.001 | 6.18 | 2 | -20 | 70 | Supp_Motor_Area_R |
|  | < 0.001 | < 0.001 | 5.82 | -2 | 28 | 72 | Paracentral_Lobule_R |
| 104 | < 0.001 | 0.135 | 4.17 | 50 | -28 | 24 | SupraMarginal_R |
| 38 | < 0.001 | 0.259 | 3.92 | -48 | -4 | 48 | Precentral_L |
| 24 | < 0.001 | 0.725 | 3.37 | -54 | -34 | 18 | SupraMarginal_L |
| **Post-treatment > Pre-treatment in the NTD group** | | | | | | | |
| 21 | < 0.001 | 0.443 | 3.68 | 22 | -36 | -12 | ParaHippocampal_R |
| 13 | 0.001 | 0.697 | 3.40 | 10 | -56 | -2 | Lingual_R |
|  | 0.001 | 0.744 | 3.35 | 5 | -48 | -10 | Vermis IV–V |
| 8 | 0.001 | 0.750 | 3.34 | -6 | -46 | -10 | Cerebelum IV–V_L |
| **Post-treatment > Pre-treatment in the TD group** | | | | | | | |
| 32 | < 0.001 | 0.134 | 4.17 | -34 | -38 | 64 | Postcentral_L |
| 13 | < 0.001 | 0.360 | 3.78 | -14 | -16 | 0 | Thalamus_L |
| 37 | < 0.001 | 0.657 | 3.45 | 24 | -36 | 8 | Hippocampus_R |
| 28 | < 0.001 | 0.667 | 3.44 | 12 | -22 | 2 | Thalamus_R |
| 34 | < 0.001 | 0.671 | 3.43 | 30 | -24 | 62 | Precentral_R |
| 8 | < 0.001 | 0.707 | 3.39 | 46 | 2 | -6 | Insula_R |

**Supplementary Table 4. Changes in functional connectivity after rehabilitation during the foot tapping task**

| **Post-treatment > Pre-treatment in the NTD group** | | | |
| --- | --- | --- | --- |
| Regions | Regions | T value | P value |
| Frontal_Inf_Tri_L | Rectus_R | 3.39 | < 0.001 |
| Thalamus_R | Cerebelum Crus Ⅰ_R | 3.37 | < 0.001 |
| Cerebelum Ⅲ_R | Vermis IV–V | 3.34 | < 0.001 |
| **Post-treatment < Pre-treatment in the NTD group** | | | |
| ParaHippocampal_L | Temporal_Pole_Mid_R | - 3.66 | < 0.001 |
| Calcarine_R | Cerebelum Crus Ⅱ_L | - 3.72 | < 0.001 |
| Fusiform_L | Pallidum_R | - 3.31 | < 0.001 |
| Putamen_L | Cerebelum Crus Ⅱ_L | - 3.39 | < 0.001 |
| **Post-treatment > Pre-treatment in the TD group** | | | |
| Rectus_L | Thalamus_L | 4.07 | < 0.001 |
| **Post-treatment < Pre-treatment in the TD group** | | | |
| Olfactory_L | Temporal_Pole_Mid_L | - 4.18 | < 0.001 |
| Cingulum_Ant_L | Temporal_Pole_Sup_R | - 4.11 | < 0.001 |
| Cingulum_Ant_R | Temporal_Pole_Sup_L | - 3.91 | < 0.001 |
| Precuneus_L | Cerebelum Ⅹ_L | - 4.26 | < 0.001 |

**Supplementary Figure Legends**

**
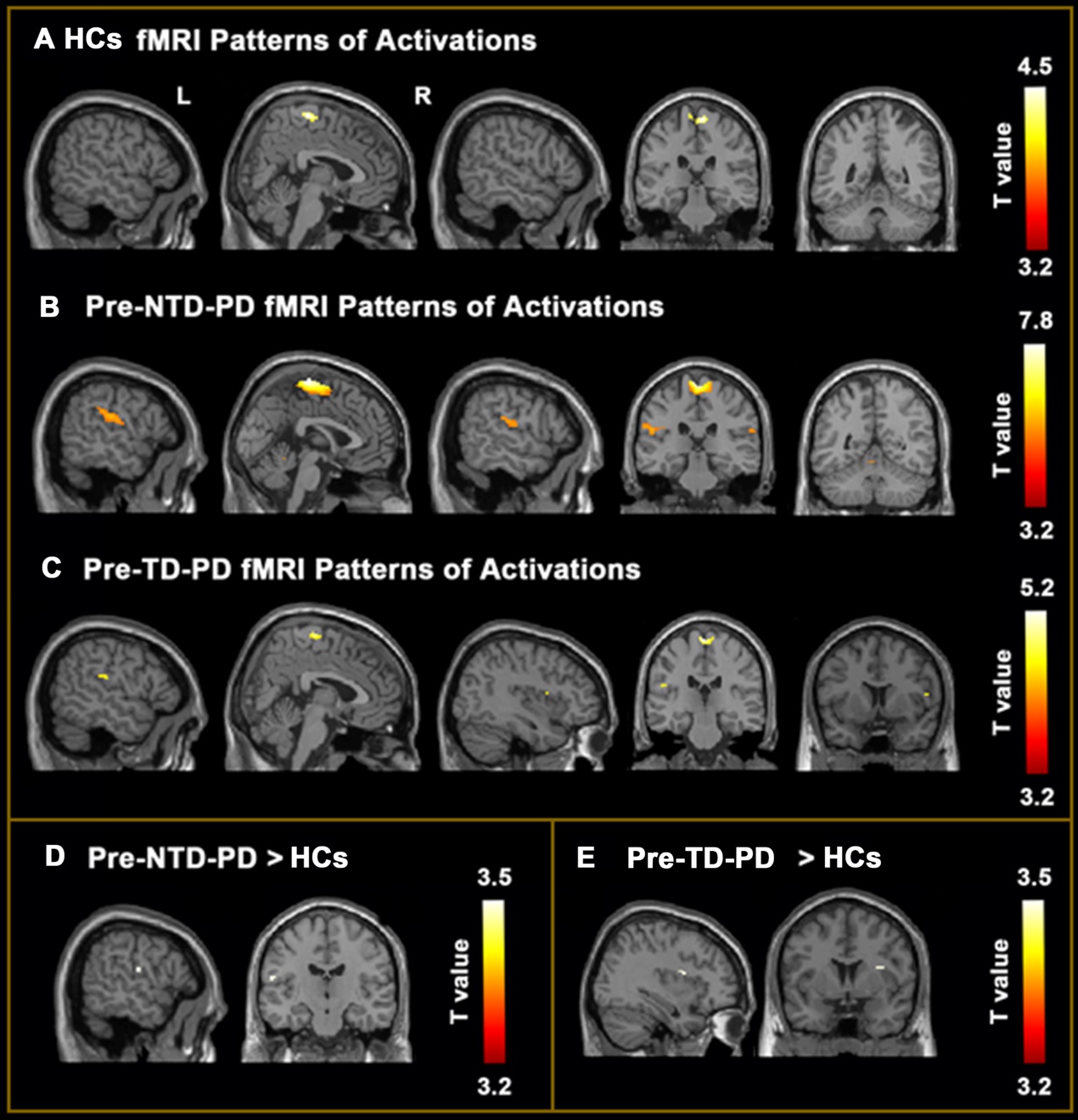
**

**Supplementary Fig. 1** fMRI patterns of activations on a rendered brain in HCs and PWPs during the foot tapping task at baseline. Brain activity during the foot tapping task in HCs **(A)**, NTD-PD patients **(B)**, and TD-PD patients **(C).** **D** fMRI differences in NTD-PD patients relative to HCs. **E** fMRI differences in TD-PD patients compared to HCs. All results are shown at P < 0.001 uncorrected, and only clusters greater than 5 voxels are reported. Color bars denote T values. HCs, healthy controls; TD, tremor-dominant; NTD, nontremor-dominant; L, the left hemisphere; R, the right hemisphere.


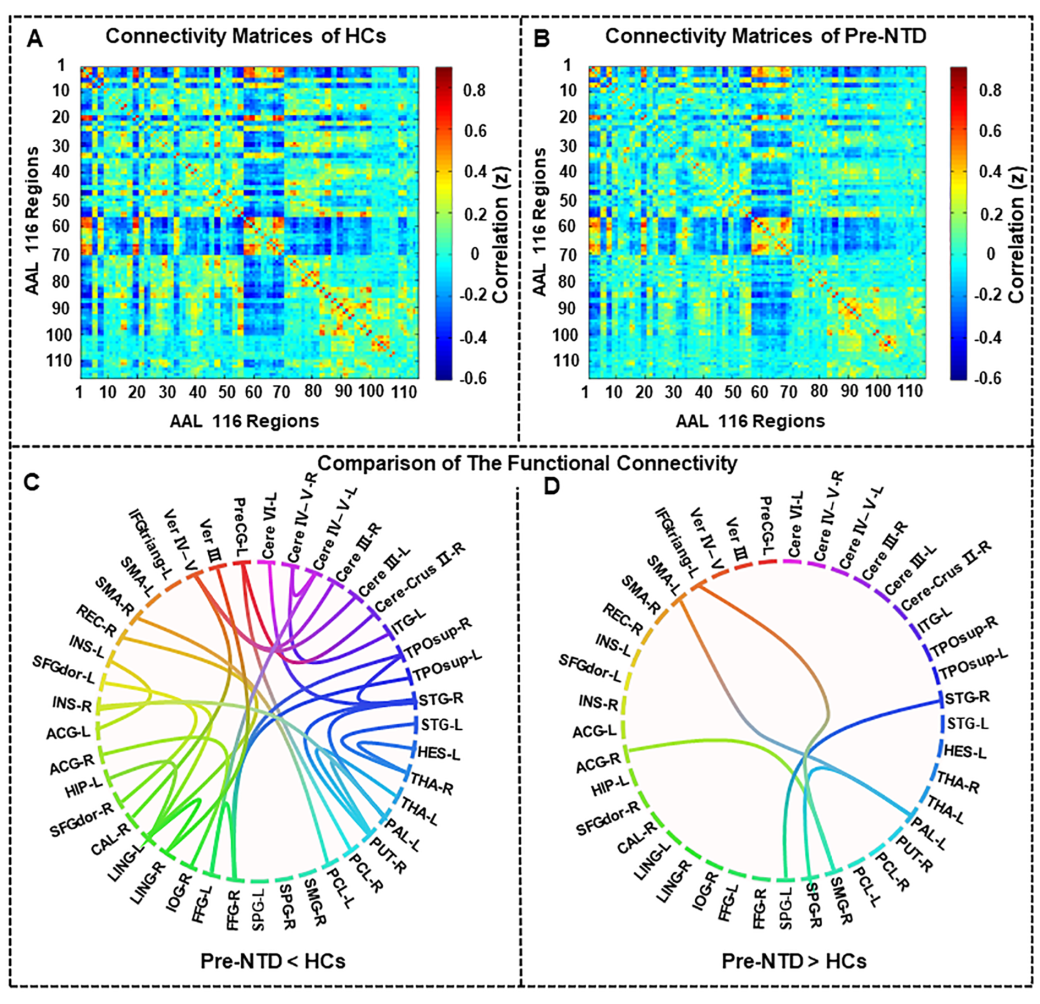


**Supplementary Fig. 2** Functional connectivity of HCs, NTD-PD patients and between-group differences at baseline. Whole-brain task-based functional connectivity matrices of HCs **(A)** and NTD-PD patients **(B)**. Connectivity values in the connectivity matrices represent the Fisher’s z-transformed Pearson correlation coefficient, averaged over all subjects. The circle plots display differences between NTD-PD patients > HCs **(C)** as well as NTD-PD patients < HCs **(D)**. All results are shown at P < 0.001 uncorrected. HCs, healthy controls; NTD, nontremor-dominant; AAL, automated anatomical labeling; Cere, the cerebellum; ITG, the inferior temporal gyrus; TPOsup, the superior temporal gyrus of the temporal pole; STG, the superior temporal gyrus; HES, the Heschl gyrus; THA, the thalamus; PAL, the pallidum; PUT, the putamen; PCL, the paracentral lobule; SMG, the supramarginal gyrus; SPG, the superior parietal gyrus; FFG, the fusiform gyrus; IOG, the inferior occipital gyrus; LING, the lingual gyrus; CAL, the calcarine fissure and surrounding cortex; SFGdor, the dorsolateral superior frontal gyrus; HIP, the hippocampus; ACG, the anterior cingulate and paracingulate gyri; INS, the insula; REC, the gyrus rectus; SMA, the supplementary motor area; IFGtriang, the triangular part of the inferior frontal gyrus; Ver, the vermis; PreCG, the precental gyrus; L, the left hemisphere; R, the right hemisphere.


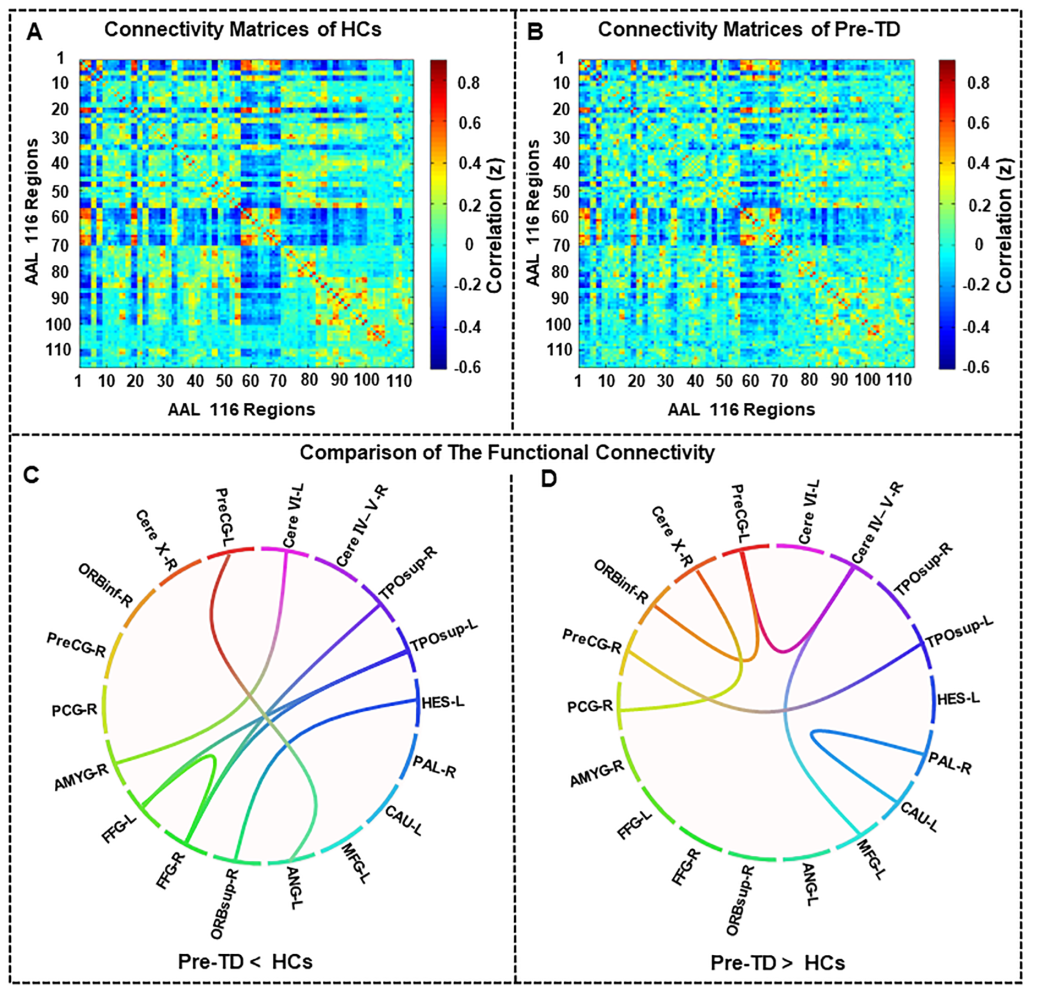


**Supplementary Fig. 3** Functional connectivity of HCs, TD-PD patients and between-group differences at baseline. Whole-brain task-based functional connectivity matrices of HCs **(A)** and TD-PD patients **(B)**. Connectivity values in the connectivity matrices represent the Fisher’s z-transformed Pearson correlation coefficient, averaged over all subjects. The circle plots display differences between TD-PD patients > HCs **(C)** as well as TD-PD patients < HCs **(D)**. All results are shown at P < 0.001 uncorrected. HCs, healthy controls; TD, tremor-dominant; AAL, automated anatomical labeling; Cere, the cerebellum; TPOsup, the superior temporal gyrus of the temporal pole; HES, the Heschl gyrus; PAL, the pallidum; CAU, the caudate nucleus; MFG, the middle frontal gyrus; ANG, the angular gyrus; ORBsup, the orbital part of superior frontal gyrus; FFG, the fusiform gyrus; AMYG, the amygdala; PCG, the posterior cingulate gyrus; PreCG, the precental gyrus; ORBinf, the orbital part of inferior frontal gyrus; L, the left hemisphere; R, the right hemisphere.


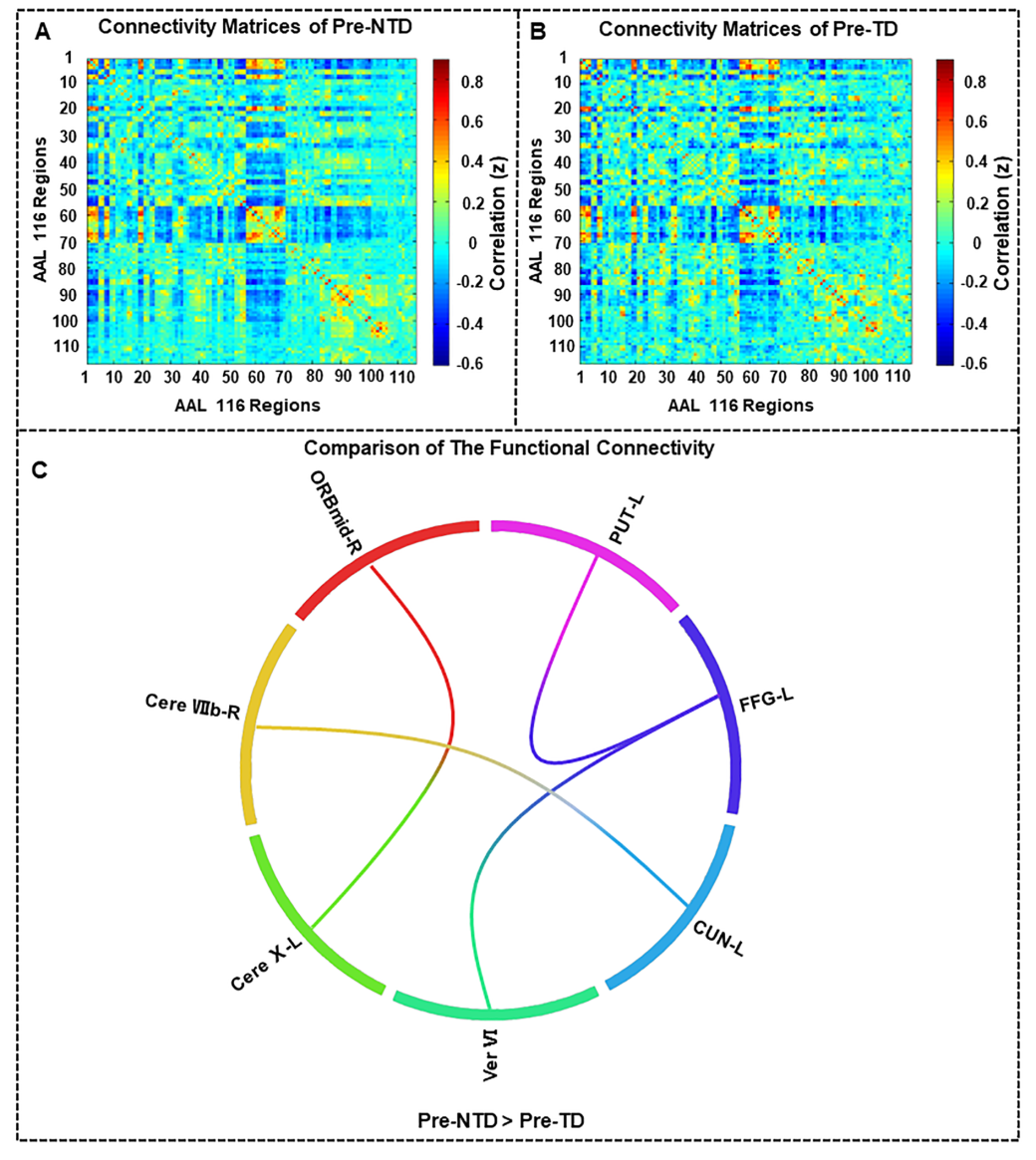


**Supplementary Fig. 4** Functional connectivity of NTD-PD, TD-PD patients and between-group differences at baseline. Whole-brain task-based functional connectivity matrices of NTD-PD (A) and TD-PD patients (B). Connectivity values in the connectivity matrices represent the Fisher’s z-transformed Pearson correlation coefficient, averaged over all subjects. The circle plots display differences between NTD-PD patients > TD-PD patients (C). All results are shown at P < 0.001 uncorrected. NTD, nontremor-dominant; TD, tremor-dominant; AAL, automated anatomical labeling; ORBmid, the orbital part of middle frontal gyrus; Cere, the cerebellum; Ver, the vermis; CUN, the cuneus; FFG, the fusiform gyrus; PUT, the putamen; L, the left hemisphere; R, the right hemisphere.
