# Supplementary figures and images for: Rehabilitation Response in Tremor‐ and Non‐Tremor‐Dominant Parkinson Disease: A Task‐fMRI Study
Source: Brain Behav. 2024 Oct 17;14(10):e70102. doi: 10.1002/brb3.70102 (PMC11483598; doi:10.1002/brb3.70102)

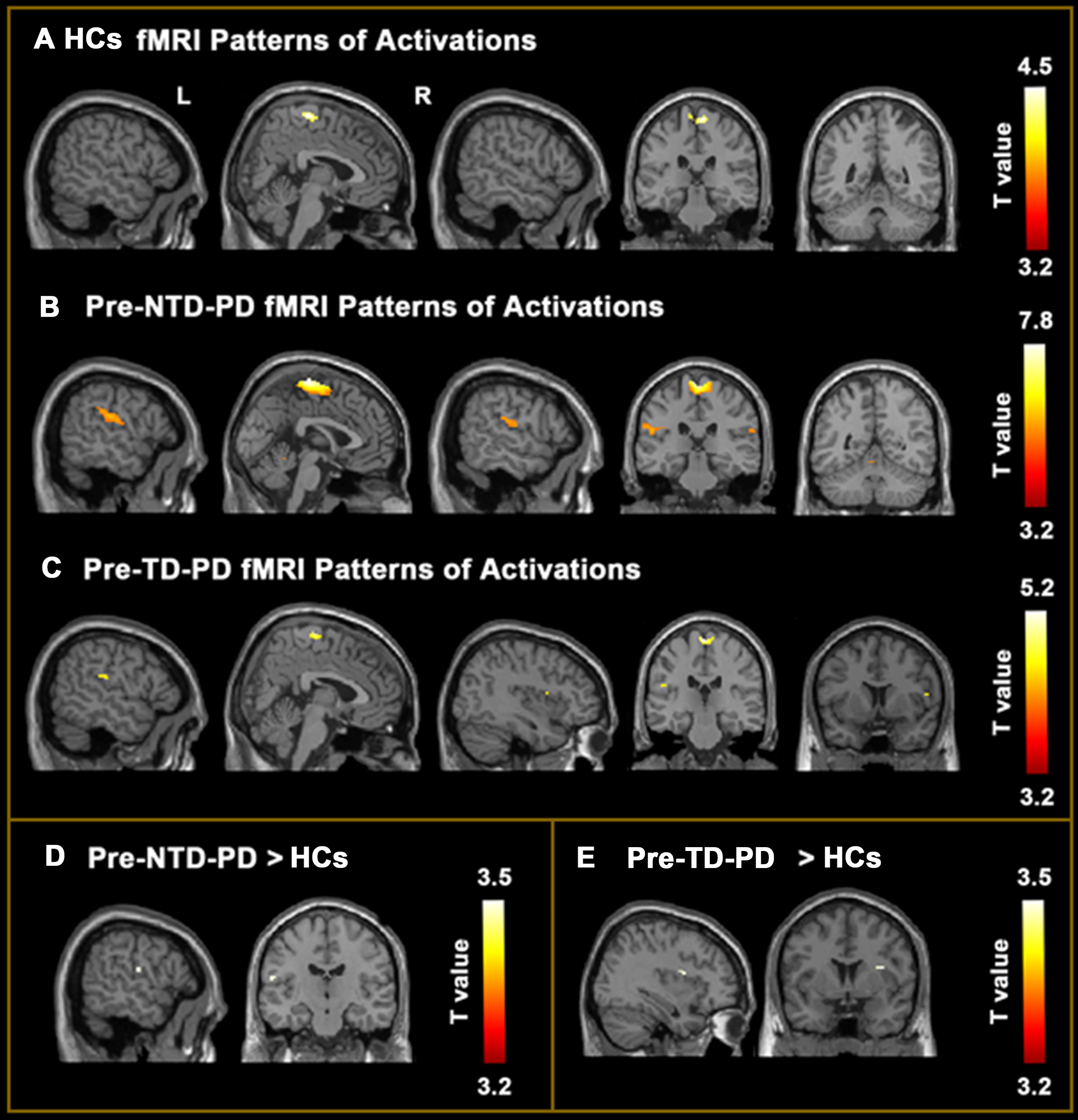

Supplement: Supplementary file 2 — Supporting Information [file BRB3-14-e70102-s001.tif]

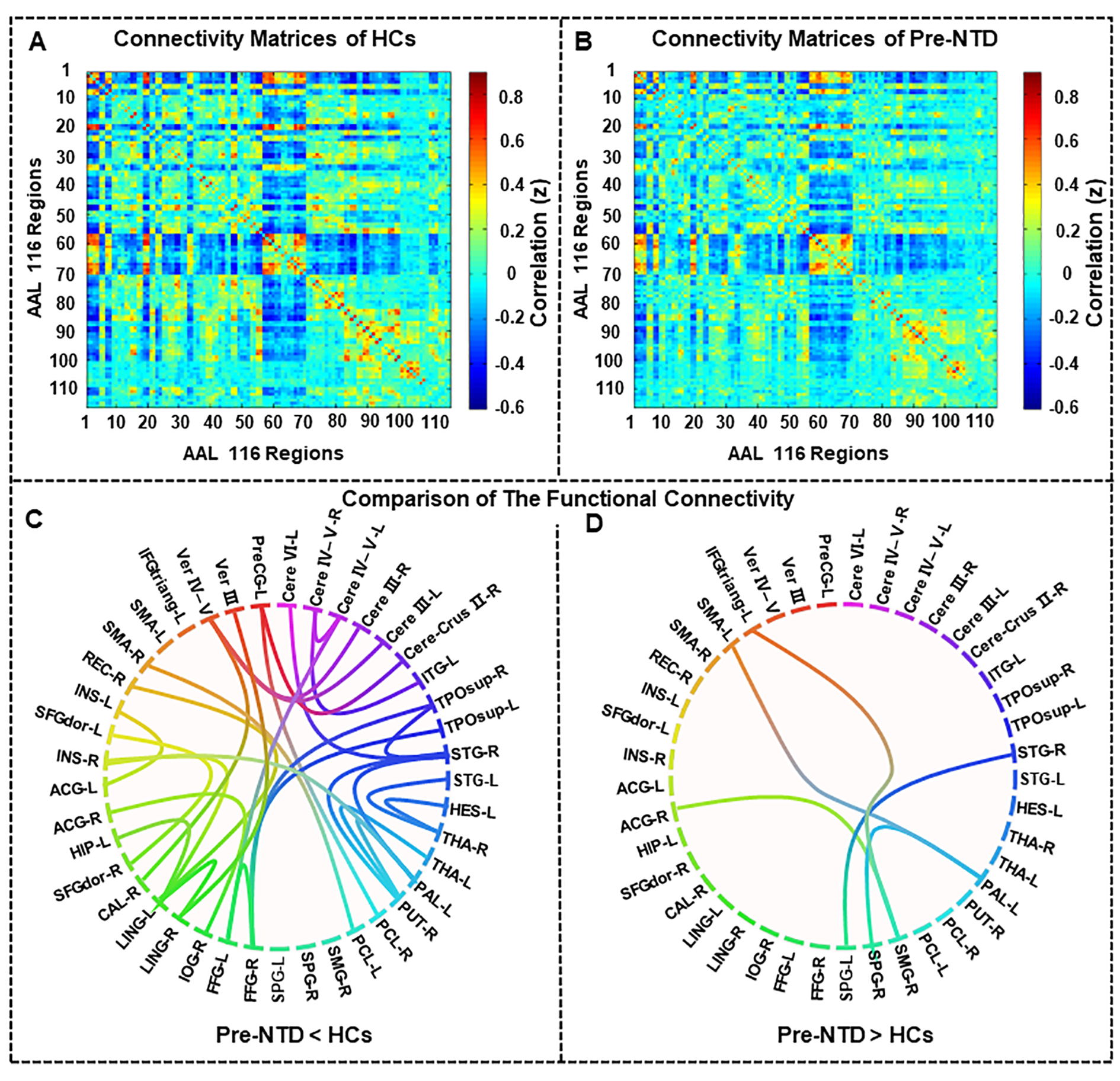

Supplement: Supplementary file 3 — Supporting Information [file BRB3-14-e70102-s003.tif]

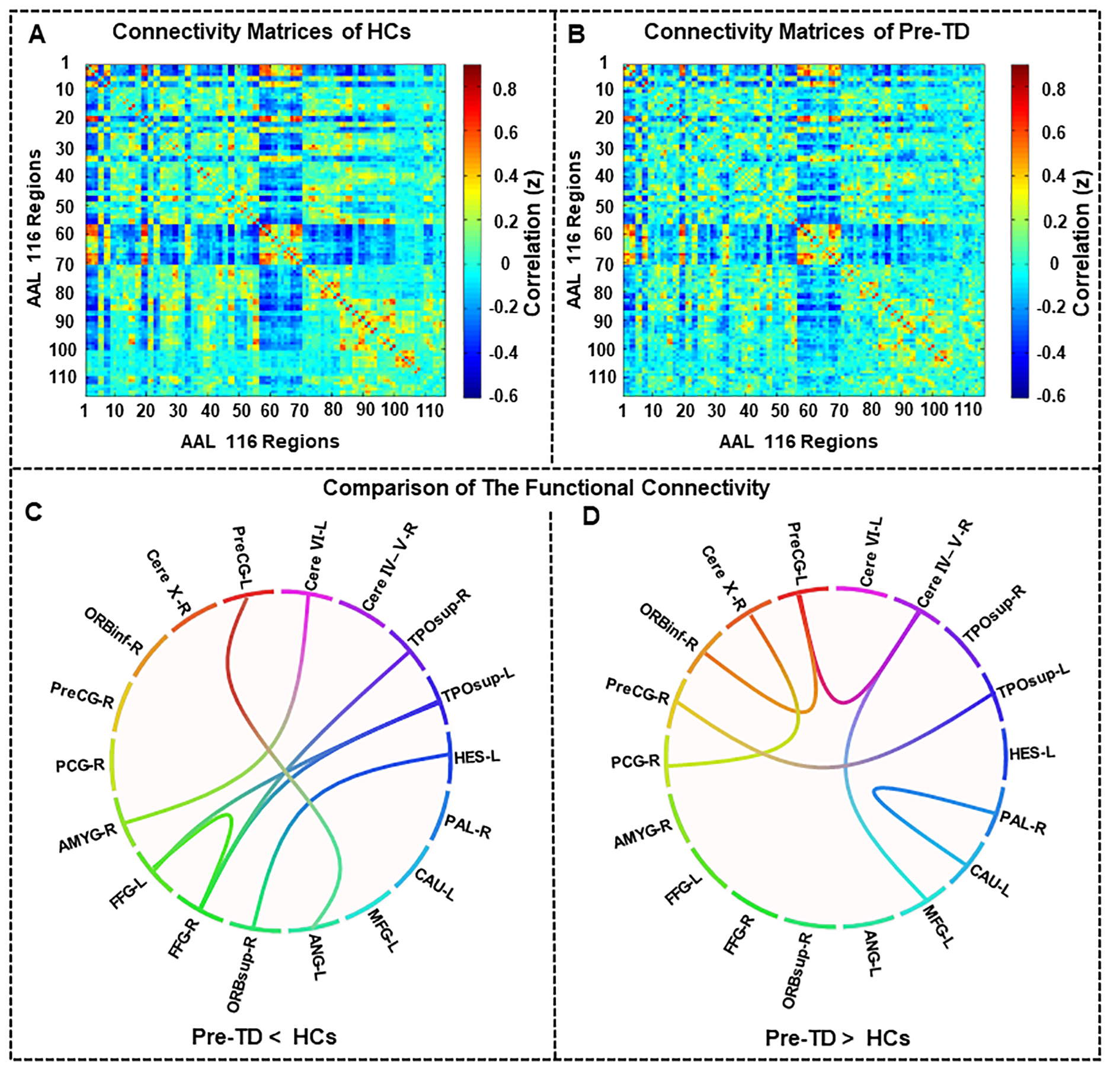

Supplement: Supplementary file 4 — Supporting Information [file BRB3-14-e70102-s005.tif]

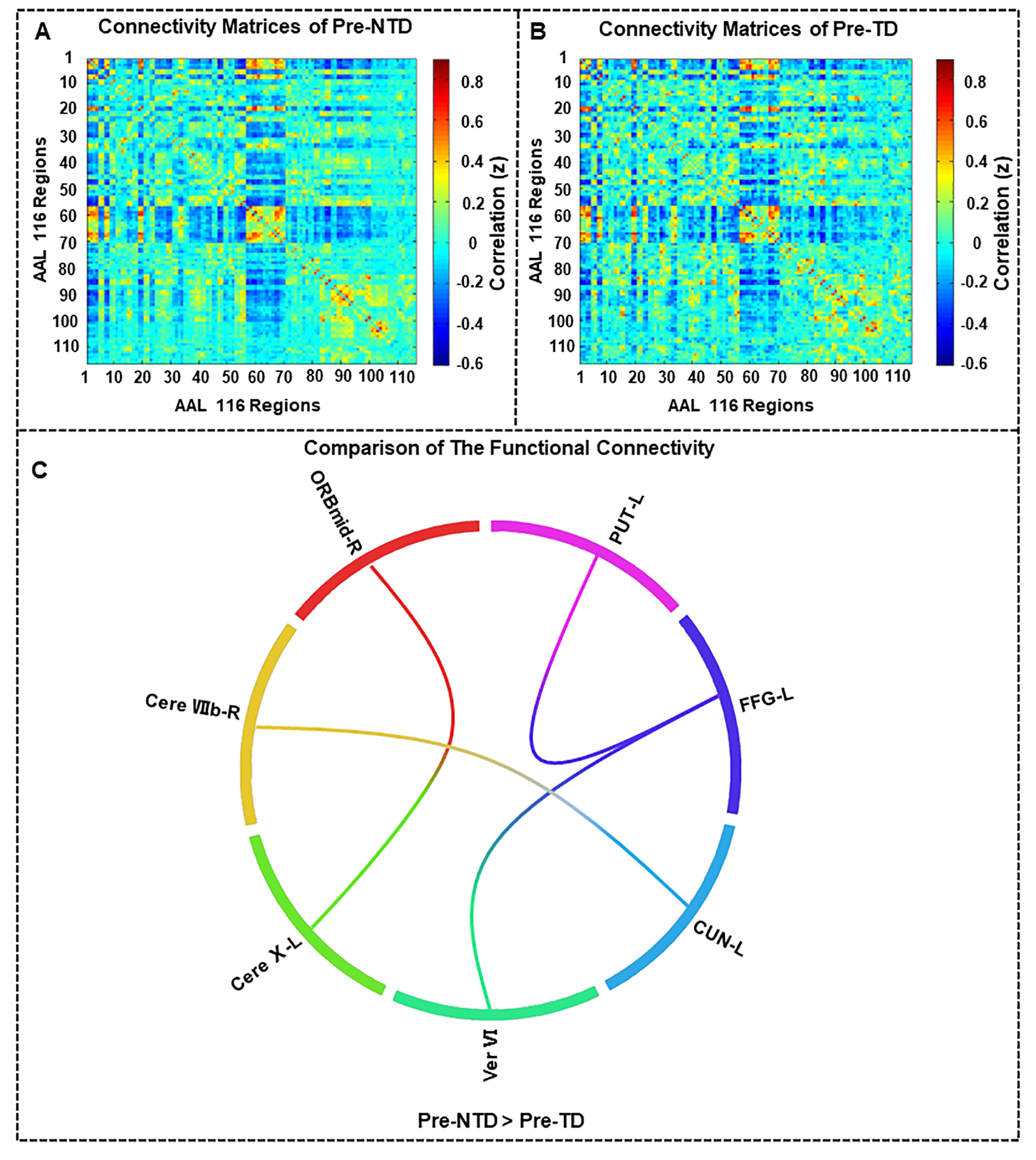

Supplement: Supplementary file 5 — Supporting Information [file BRB3-14-e70102-s002.tif]
